# Fetal pharyngeal glial heterotopia manifested as polyhydramnios: a rare case with difficult prenatal diagnosis

**DOI:** 10.1186/s12884-023-05373-3

**Published:** 2023-01-13

**Authors:** Xiaoting Ke, Huaijie Cai, Wei Wang, Qingquan Lai

**Affiliations:** 1 Department of CT/MRI, The Second Affiliated Hospital of Fujian Medical University, Quanzhou, 362000 Fujian Province China; 2 Department of Ultrasound, Quanzhou First Hospital Affiliated to Fujian Medical University, Quanzhou, 362000 Fujian Province China; 3 Department of Ultrasound, Affiliated Hangzhou First People’s Hospital, Zhejiang University School of Medicine, Hangzhou, 310006 Zhejiang Province China

**Keywords:** Glial heterotopia, Pharyngeal, Polyhydramnios, Imaging, Case report

## Abstract

**Background:**

Glial heterotopia is a rare congenital developmental malformation that presents as tumor-like lesions of the nerve tissue that grow outside the nervous system, but are not true tumors. At present, most cases are reported in neonates and children and are very rarely found in fetuses. The present report describes a case of fetal pharyngeal glial heterotopia and associated imaging findings to better understand the disease in the future.

**Case presentation:**

A 32-year-old pregnant woman was admitted to the hospital with polyhydramnios. An ultrasound examination revealed a hypoechoic mass in the neck of the fetus. Magnetic resonance imaging showed a well-defined mass with significant compression of the esophagus and airway. The amniotic fluid index was approximately 40 cm. Considering that difficulty swallowing and breathing may occur due to compression by the mass after birth, tracheotomy and mass resection should be performed immediately. The difficulty of the tumor resection procedure and the nature of the tumor are both factors affecting the prognosis of the fetus. The pregnant woman eventually chose to induce labor. The fetal pharyngeal mass was then resected and its pathological examination indicated pharyngeal glial heterotopia.

**Conclusions:**

Polyhydramnios due to pharyngeal glial heterotopia is extremely rare and accurate prenatal diagnosis is challenging. Clinical diagnosis of glial heterotopia in preterm fetuses is difficult. Therefore, understanding glial heterotopia is helpful to improve clinical treatment options.

## Background

Glial heterotopia is a rare congenital developmental abnormality [1]. It typically presents as a tumor-like lesion, although it is not a true tumor, and occurs when the nerve tissue is found outside of the central nervous system. Glial heterotopia is diagnosed in neonates and children, with the most common occurrence in the middle line of the nose, scalp, orbit, palate, and other areas [2, 3]. There is no special clinical manifestation of glial heterotopia, which is related to the location and size of the mass. Some patients are diagnosed due to respiratory tract compression. Surgical resection is the treatment for glial heterotopia. Since it is not a true tumor, its prognosis is satisfactory without postoperative recurrence [1]. However, pharyngeal glial heterotopia in the fetus is extremely rare. To the best of our knowledge, only one case has been reported so far [4]. An ultrasound examination can dynamically observe the position and size of the mass, amniotic fluid, and umbilical cord blood flow during pregnancy. Magnetic resonance imaging (MRI) is a better imaging method for evaluation of the mass location, adjacent tissue, and glial heterotopia composition [5]. The present report describes a case of a 32-year-old woman presenting with hydramnios who was discovered to have a fetal pharyngeal mass after further examination. Prenatal diagnosis was challenging because this condition is very rare. Based on the present case and a review of previous literature, the imaging findings and clinical decisions associated with glial heterotopia were explored in order to improve our understanding of the disease.

## Case presentation

A 32-year-old pregnant woman at 34 weeks of gestation was admitted to the hospital due to polyhydramnios lasting for 11 days that was diagnosed via examination. The pregnant woman was healthy in the past and without any history of surgery or drug allergy. She had regular outpatient prenatal examinations and no obvious abnormality was found in the fetus during early and middle pregnancy. An ultrasound examination indicated an amniotic fluid index of 38–40 cm (normal range is about 8–18 cm) and a maximum amniotic fluid depth of 17 cm 11 days before admission.

**Fig. 1:**
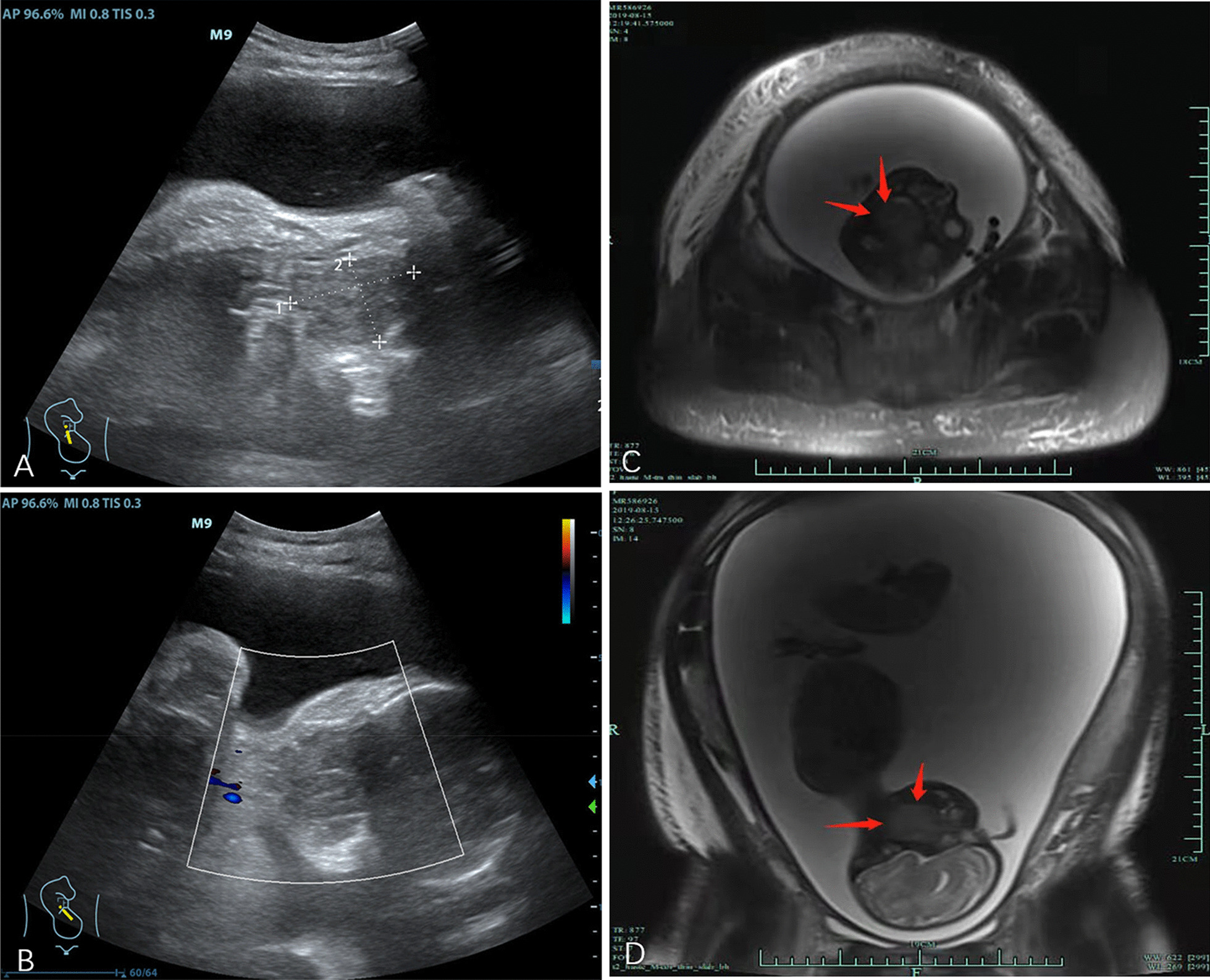
Ultrasound and MRI images of pharyngeal glial heterotopia. **A, B** Sonographic examination demonstrating a slightly hypoechoic mass (5.4 cm × 4.4 cm × 3.4 cm) in the pharynx of the fetus with a clear boundary and without detectable blood flow. **C**, **D** Pelvic magnetic resonance imaging (MRI) revealed a solid mass (4.5 cm × 3.7 cm × 3.3 cm) with a complete capsule and slight hyperintensity on T2WI in the fetal pharynx

There were no positive routine examination results after admission. The obstetric examination results were as follows: uterine height of 50 cm, abdominal circumference of 111 cm, fetal heart of 150 times/min, fetal position ROA, head exposure, no contractions, closed uterine orifice, and intact membranes. Relevant data from the ultrasonic examination were as follows: double parietal diameter of 8.2 cm, head circumference of 30.0 cm, abdominal circumference of 28.1 cm, femur length of 6.5 cm, amniotic fluid index of 40 cm, and cord blood flow systolic/diastolic blood pressure of 3.5. The size of the fetus was about 34 weeks, which was close to the actual gestational age. Fetal gastric vesicles were faintly visible at examination, with a range of about 1.4 cm × 1.1 cm × 0.7 cm and low tension. A slightly hypoechoic mass was observed in the pharynx of the fetus. It had a clear boundary and a size of about 5.4 cm × 4.4 cm × 3.4 cm (Fig. 1A, B). The amniotic fluid index was about 40–42 cm. At the same time, the maternal renal pelvis was slightly hydronephrotic and about 1.0 cm wide. Routine urine testing results suggested proteinuria (++). The patient also suffered from hydronephrosis due to hydramnios and the increase in intra-abdominal pressure. If the disease developed further, it may have caused maternal organ dysfunction and even abdominal space syndrome. A pelvic MRI was performed in order to further understand the nature and composition of the mass.

**Fig. 2:**
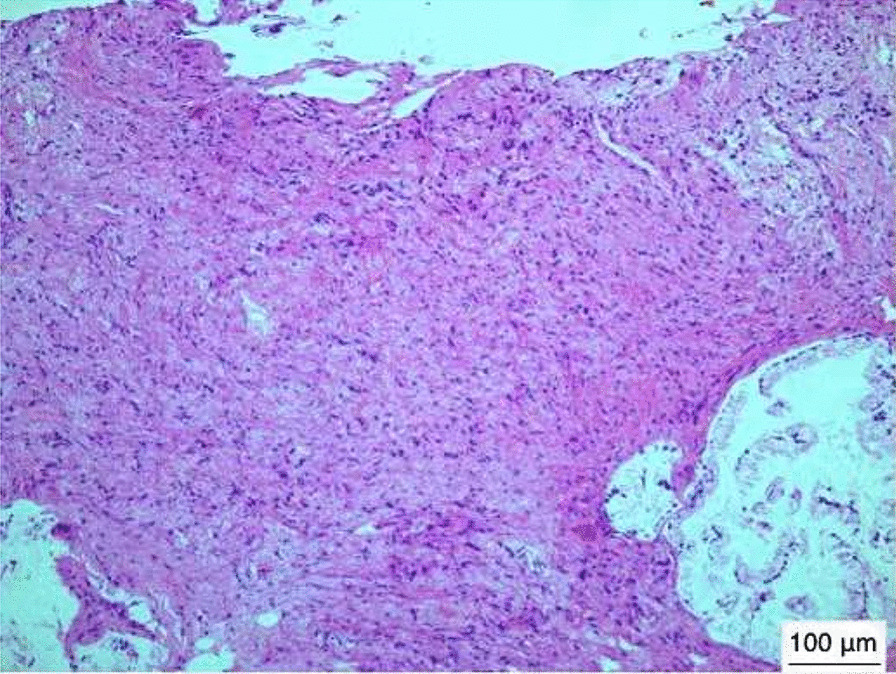
Histologic examination showing abundantly glial cell composition, with nests of GFAP-immunoreactive neuropils containing large, often multinucleated astrocytes (hematoxylin–eosin stain; microscope, OlympusBX41/×100; software, NIS-Elements F 4.60.00 64-bit; camera, Nikon DS-Fi3;scale bars, 100 μm)

**Fig. 3:**
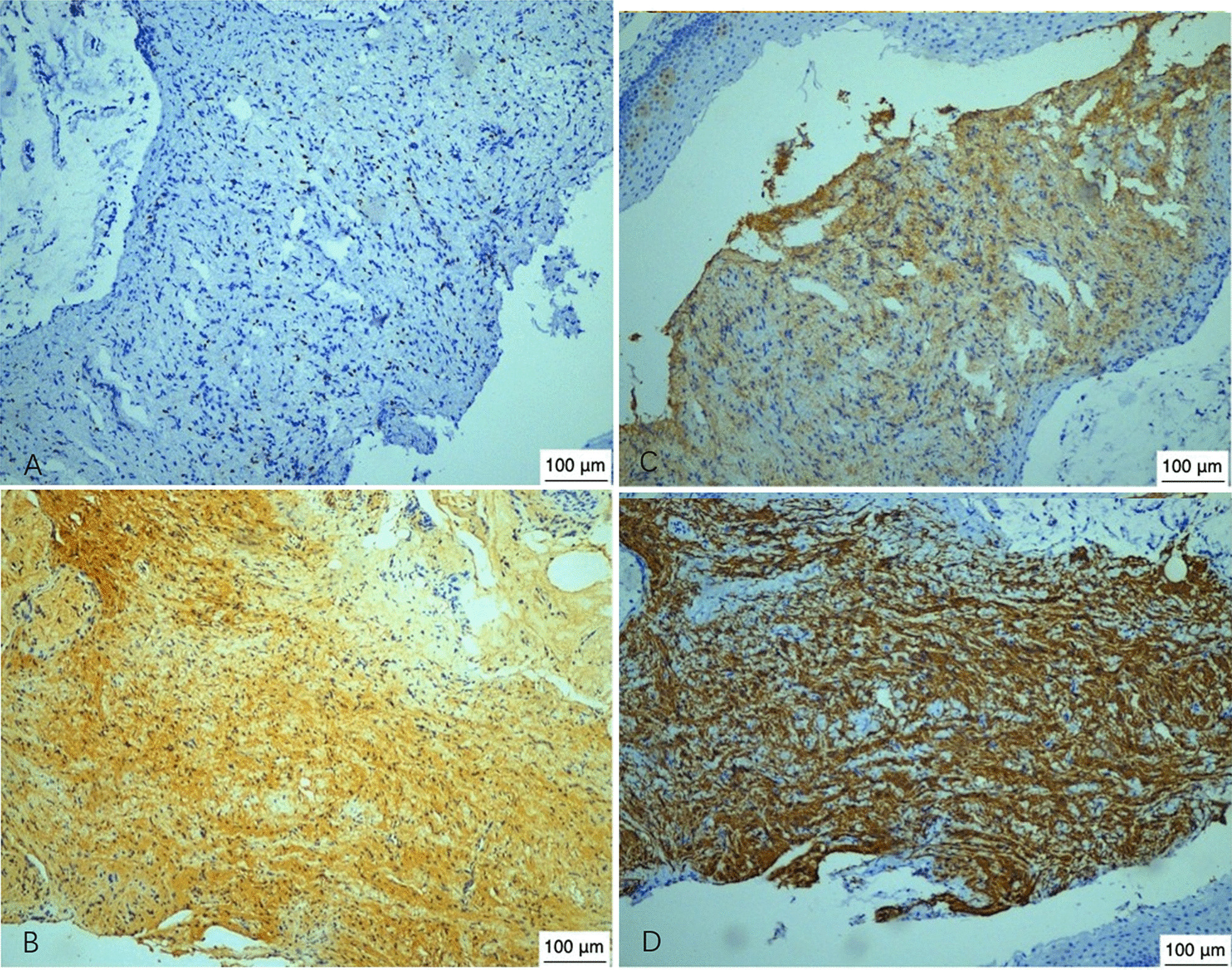
Immunohistochemical analysis. **A** Immunohistochemical analysis revealed SOX-10 [+]. **B** Immunohistochemical analysis revealed S-100 [+]. **C** Immunohistochemical analysis revealed NSE [+]. **D** Immunohistochemical analysis revealed GFAP [+]. (Hematoxylin–eosin stain; microscope, OlympusBX41/×100; software, NIS-Elements F 4.60.00 64-bit; camera, Nikon DS-Fi3;scale bars, 100 μm)

**Fig. 4:**
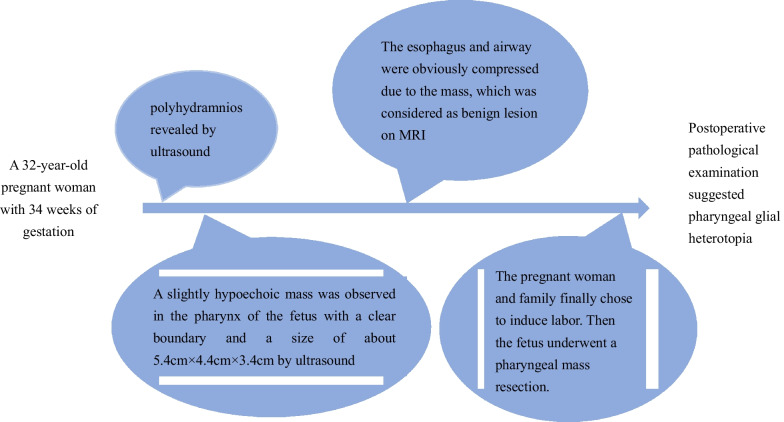
The diagnostic flow chart of antepartum pharyngeal glial heterotopia

The MRI results demonstrated a slight hyperintensity in the fetal pharynx mass on a T2WI, which was about 4.5 cm × 3.7 cm × 3.3 cm in size. The mass capsule was complete (Fig. 1C, D), reaching the top of the nasopharynx and down to the epiglottis. The esophagus and the airway were obviously compressed. The amniotic fluid had a maximum depth of 17 cm. Combined with the above-mentioned relevant examination results, it was evident that polyhydramnios was related to the fetal pharyngeal mass. Dysphagia and dyspnea may have occurred after birth due to compression by the mass. Tracheotomy and mass resection would have to be performed immediately after birth. The prognosis of the fetus depended on the nature of the mass. After a detailed consultation with the pregnant woman and her family, she decided to give up the fetus. The clinician used a puncture needle to extract an appropriate amount of amniotic fluid to relieve the symptoms of intrauterine compression and injected 100 mg of rivanol. Two days later, the pregnant woman naturally gave birth to a female stillborn fetus weighing 2,000 g and with immature appearance. An oral examination showed a lump in the pharynx, which was solid and hard in texture. A pathological examination was carried out after the excision and revealed pharyngeal glial heterotopia (Fig. 2). Immunohistochemical results were as follows: SOX-10 [+], S-100 [+], NSE [+], GFAP [+], SMA [−], Ki67 [+], mucous gland CK7 [+], CK20 [−], and special staining: AB [−], PAS [+] (Fig. 3). The process of discovery and diagnosis of this case is shown in the figure (Fig. 4).

## Discussion and conclusions

Glial heterotopia is a rare congenital disease. It is not a true tumor, but rather glial tissue located outside the central nervous system. It has previously been called glial hamartoma, brain heterotopia, and glial branching tumor [6]. At present, the pathogenesis of glial heterotopia remains unclear. However, there are three main hypotheses that attempt to explain it. In the first hypothesis, the descending brain tissue bulges outward through the defective skull. Once the defective skull is closed, the bulging brain tissue eventually becomes glial heterotopia [7]. In the second hypothesis, the displacement of neuroectodermal cells is caused by the failure of anterior foramen closure. Abnormal migration of olfactory bulb glial cells then leads to glial heterotopia [8]. There are mature and immature types of ectopic glia, which are mainly composed of neurons, meningeal tissue, choroid tissue, and ependymal epithelial cells in different proportions [9].

Based on statistical data, glial heterotopia is typically diagnosed in newborns and infants. The nasal cavity is the most common tumor origin, and its clinical manifestations depend on tumor size and location. Most cases are discovered after birth based on body surface mass or airway obstruction. Some patients with exogenous glial heterotopia are often misdiagnosed with hemangioma because of reddish or purple skin color. Glial heterotopia occurs deep in the tissue and remains hidden. It is usually noticed after the tumor grows enough to cause the surrounding organs to be compressed.

The prenatal diagnosis of glial heterotopia is extremely rare. Ferraz-Filho et al. [4] have reported a 25-week-old fetus diagnosed with exogenous pharyngeal glial heterotopia, where the tumor did not grow during the follow-up of 11 weeks. The mother underwent a cesarean section at week 36, and the Apgar scores of the newborn at 1 and 5 min were 9 and 10, respectively. The newborn was sent to the intensive care unit for observation after mechanical ventilation was performed. The child’s glial heterotopia was successfully removed two months later and there was no recurrence or growth retardation noted at the 4-year follow-up.

Looking back at our case, no abnormal mass was found in the pharynx of the fetus during the first and second trimesters. An indirect sign of polyhydramnios was discovered in the third trimester. However, its causes were complicated and unclear, and only some of them have been previously reported. Generally speaking, common causes include fetal malformation and chromosomal abnormalities, such as fetal nervous system defects, digestive system defects (esophageal atresia, duodenal atresia), abdominal wall defects, diaphragmatic hernia, frontal malformation, urinary system malformation, chromosomal abnormalities (trisomy 18, 21, and 13), and maternal factors, such as gestational diabetes mellitus and idiopathic polyhydramnios. In addition, the stomach bubble tension in the fetus is low, so that the clinical diagnosis tends to be that of fetal digestive tract malformation. Finally, the upper digestive tract of the fetus was carefully explored again before the pharyngeal lump was found. MRI findings suggested benign lesions, but there were many uncertain factors due to the rarity of the fetal neck masses and the complexity and diversity of developmental malformations. Common neck masses include lymphatic deformity, teratoma, branchial anomaly, and vascular deformity [10]. Moreover, the mass in this case exerted pressure on the esophagus and airway of the fetus. In order to save the fetus, it was necessary to perform a tracheotomy immediately after the cesarean section to ensure the respiratory circulation of the fetus. The pharyngeal mass in the fetus then had to be removed. In the end, the pregnant woman gave up the fetus and chose to induce labor. Postoperative pharyngeal mass was diagnosed as glial heterotopia via pathological examination. Since fetal death due to the lack of knowledge about glial heterotopia is not a desirable outcome, it is particularly important to fully understand the imaging features of glial heterotopia for prenatal diagnosis, which is helpful to formulate treatment strategies and deal with fetal respiratory distress and feeding difficulties after birth [11]. Moreover, glial heterotopia may also be combined with other deformities, such as cleft palates, Pierre Robin syndrome, congenital heart disease, pectus excavatum, and micrognathia [12], which cannot be ignored. Based on previous reports, glial heterotopia is a round or quasi-round solid mass, and its computerized tomography (CT) density and MRI signal are related to the maturity of heterotopic nerve tissue. The components of pharyngeal glial heterotopia can be solid, cystic, or cystic-solid, and the solid signal or density is similar to that of cerebral gray matter. For the solid mass, the CT results generally show that the mass boundary is clear, while contrast enhancement is uniform. In addition, a CT scan can clearly show the relationship between the lesion and the adjacent bone tissue. Imaging diagnosis can distinguish glial heterotopia from other tumors by the presence or absence of bone destruction, because most glial heterotopias are characterized by bone compression. Due to the high resolution of the soft tissue images, MRI can show the location of the lesion and the surrounding tissue more clearly and evaluate the compression of the trachea and esophagus. It also shows that the T1WI signal is similar to that of gray matter, while the T2WI signal is slightly higher [13], with a clear boundary with surrounding soft tissues, no edema present, slight uniform enhancement of the lesion, and more obvious edge on contrast enhancement. However, some scholars believe that ectopic nerve tissue dysplasia is usually not enhanced, while the compressed tissue can be slightly enhanced [6]. Whether glial heterotopia is enhanced or not may be related to the fibrous blood vessels [3]. For cystic components, glial heterotopia can be single or multi-cystic. Contrast enhancement shows that the cystic wall is slightly or obviously enhanced, which is similar to a lymphatic malformation [14]. Pathology analysis has shown that the cystic component is actually cerebrospinal fluid, which is produced by the functional choroidal plexus in ectopic glial tissue. A prior study has shown that the thinning or erosion of the middle cranial fossa and adjacent bones under pressure is often a characteristic manifestation of glial heterotopia [15], which can be used as a sign to differentiate it from a lymphatic malformation. In the present case, glial heterotopia presented as a solid mass, and the MRI findings were consistent with the description of previous studies. It was also preliminarily diagnosed as a benign lesion. Pharyngeal masses can be diagnosed based on combined relevant ultrasound and MRI findings. Ultrasound is a simple and non-radiation tool that is particularly important for prenatal diagnosis of glial heterotopia. It can help to observe the location and size of the mass and other malformations, which plays a very important role in fetal monitoring. In the present case, both the ultrasound and MRI examinations played an important role in the preliminary diagnosis of glial heterotopia. Therefore, ultrasound and MRI can complement each other in prenatal imaging diagnosis of glial heterotopia.

Surgical resection can be used for the treatment of glial heterotopia and the recurrence probability then becomes extremely low. Because it is not a true tumor, the long-term prognosis is satisfactory. Therefore, the advantages of different imaging examinations and their related features need to be fully understood, especially in prenatal glial heterotopia. Different diagnoses and decisions may have completely different results. In the present case, if the fetus was allowed to survive and a detailed treatment strategy was formulated for the pharyngeal glial heterotopia, a satisfactory outcome may have occurred.

Prenatal diagnosis of glial heterotopia has some challenges. The imaging manifestations of glial heterotopia are benign tumor-like changes, and pharyngeal glial heterotopia can be diagnosed exclusively via ultrasound and MRI examinations. Although the disease is extremely rare, clinicians need to further understand the main features of the disease, thereby reducing the possibility of misdiagnosis and improving the odds of a good prognosis.

## Data Availability

The datasets used and/or analyzed during the current study are available from the corresponding author on reasonable request.

## Abbreviations

MRI: Magnetic resonance imaging
ROA: Right occiput snterior
T2WI: T2 weighted imaging
CT: Computerized tomography
